# Development of IgY-Based Passive Immunization Against Tilapia Lake Virus: Development and In Vitro Neutralization Assays

**DOI:** 10.3390/v17030448

**Published:** 2025-03-20

**Authors:** Piyathip Setthawong, Jidapa Yamkasem, Matepiya Khemthong, Puntanat Tattiyapong, Pornphimon Metheenukul, Noppadol Prasertsincharoen, Tuchakorn Lertwanakarn, Naris Thengchaisri, Win Surachetpong

**Affiliations:** 1 Department of Physiology, Faculty of Veterinary Medicine, Kasetsart University, Bangkok 10900, Thailand; piyathip.s@ku.th (P.S.); tuchakorn.l@ku.th (T.L.); 2 Department of Veterinary Microbiology and Immunology, Faculty of Veterinary Medicine, Kasetsart University, Bangkok 10900, Thailand; jidapa.yam@ku.th (J.Y.); matepiya.kh@ku.th (M.K.); been_best@yahoo.com (P.T.); 3 Department of Veterinary Technology, Faculty of Veterinary Technology, Kasetsart University, Bangkok 10900, Thailand; pornphimon.m@ku.th (P.M.); noppadol.p@ku.th (N.P.); 4 Department of Companion Animal, Faculty of Veterinary Medicine, Kasetsart University, Bangkok 10900, Thailand; ajnaris@yahoo.com; 5 Laboratory of Biotechnology, Chulabhorn Research Institute, Bangkok 10210, Thailand

**Keywords:** tilapia lake virus, passive immunization, immunoglobulin, IgY, chicken eggs, disease management

## Abstract

Tilapia lake virus (TiLV) poses a major threat to global tilapia aquaculture and contributes to significant economic losses due to the absence of effective vaccines and treatments. Given the high mortality rates and severe pathological effects of TiLV on tilapia, alternative strategies, such as immunoglobulin-based therapies, are being considered for disease control. In this study, we developed specific immunoglobulin Y (IgY) antibodies against TiLV and evaluated their neutralization activity. Laying hens were immunized via intramuscular injections of recombinant TiLV segment 4 protein, and IgY antibodies were extracted and purified from their egg yolks using polyethylene glycol precipitation. Western blot analysis confirmed the specificity of the IgY, which demonstrated no cross-reactivity with nontarget proteins. Neutralization assays revealed a dose-dependent reduction in TiLV infectivity, which declined from 5.01 × 10^6^ TCID_50_/mL to 5.01 × 10^4^–1.26 × 10^5^ TCID_50_/mL, with the highest efficacy observed at a 1:2 dilution. Despite the variability in neutralization infectivity among the different hens, IgY effectively inhibited TiLV-induced cytopathic effects. Immunofluorescence assays further confirmed a significant reduction in the TiLV antigen levels in IgY-treated RHTiB cells. Our findings highlight IgY as a promising strategy for TiLV control and suggest its potential application in the prevention of emerging viruses.

## 1. Introduction

Since its emergence in 2014, tilapia lake virus (TiLV) has posed a significant threat to global tilapia aquaculture. TiLV causes disease across various tilapia species, including wild *Sarotherodon galilaeus*, farmed *Oreochromis niloticus*, and commercially hybrid tilapia (*O. niloticus* × *O. aureus*), with markedly adverse economic and ecological impacts [1,2]. TiLV, a segmented RNA virus with 10 genome segments [3], was recently classified under the family *Amnoonviridae* [4] and recognized as a notifiable disease by the World Organization for Animal Health [5]. Infected tilapia typically show clinical signs, such as abnormal swimming, exophthalmia, skin congestion, scale protrusion, and abdominal swelling, with mortality rates exceeding 30% within a week of the initial infection [1]. TiLV spreads through direct fish-to-fish contact [6,7], vertical transmission from infected broodstock to offspring [8,9], and environmental pathways. The virus has been detected in water where infected fish reside, but the infectivity of the virus outside the host remains to be determined [10,11]. Notably, TiLV genomic material can persist in frozen tilapia fillets for up to 28 days at −20 °C, although the likelihood of this material causing wider infection in such fillets is extremely low [12]. With its presence now reported in 18 countries, TiLV continues to be of significant concern to the global tilapia aquaculture industry [2,13].

To mitigate the impact of TiLV, researchers have explored different strategies, among them, rapid diagnostics, biosecurity measures, disinfectants, antiviral agents, the selection of fish that are genetically resistant to the virus, and vaccine development [14,15,16,17]. However, despite these efforts, no commercial vaccine is yet available, and existing interventions have failed to fully contain the spread of the virus [5,18,19]. Novel approaches, such as passive immunization using antibodies, are being explored as potential strategies to manage disease in fish farms [20] and offer promising potential for reducing the impact of TiLV. Passive immunization, which involves the administration of preformed antibodies to confer immediate protection, holds promise for preventing infections and mitigating disease severity [21,22]. However, conventional antibody production often relies on animals such as rabbits and horses and requires invasive blood collection. This practice raises ethical concerns related to animal welfare, induces stress in the animals, and presents challenges in terms of the cost effectiveness for large-scale production [23,24]. These limitations highlight the need for alternative antibody production methods that are more sustainable and ethically responsible [25,26].

Immunoglobulin Y (IgY), an immunoglobulin isotype found in birds, reptiles, and amphibians, is considered the functional equivalent of mammalian IgG [27]. IgY antibodies can be produced in large quantities by immunizing laying hens with specific antigens, followed by the extraction of the antibody from their egg yolks [28]. This approach offers several advantages, including its noninvasive nature, which minimizes animal stress, and its cost effectiveness for large-scale antibody production [22,23]. Moreover, the accumulation of IgY in egg yolks enables its easy transfer to target organisms through ingestion, thereby facilitating its use in various applications. The efficacy of IgY antibodies in preventing the replication and spread of bacterial and viral pathogens is well documented for both terrestrial and aquatic animals [29,30]. In aquaculture, passive immunization with IgY has been successfully applied to combat infectious diseases caused by pathogens, such as *Edwardsiella tarda* in Japanese eels (*Anguilla japonica*) [31], *Yersinia ruckeri* and *Vibrio anguillarum* in rainbow trout (*Oncorhynchus mykiss*) [32,33], *Pseudomonas fluorescens* and *Vibrio fluvialis* in goldfish (*Carassius auratus*) [34,35], and *Aeromonas salmonicida* in koi carp (*Cyprinus carpio koi*) [36]. Despite its proven effectiveness against bacterial pathogens, the application of IgY to address viral infections in aquaculture has been relatively limited and has not been applied to emerging viral pathogens. Previous examples include research on the use of oral IgY against cyprinid herpesvirus 3 in common carp (*Cyprinus carpio*), which reduced mortality from 50% to 85% when fish were challenged with a lethal viral dose of 40 TCID_50_/fish [37]. Furthermore, IgY has been shown to inhibit the replication of red-spotted grouper nervous necrosis virus in cell cultures and demonstrated a protective effect in vivo [38,39]. In this study, we aimed to develop and produce TiLV-specific IgY antibodies by immunizing laying hens with TiLV antigens and to evaluate their efficacy in inhibiting viral activity. The findings of this study could offer a novel and sustainable strategy for managing TiLV infections.

## 2. Materials and Methods

### 2.1. Preparation of a Recombinant TiLV-S4 Antigen

The TiLV antigen was prepared from the tissue of moribund red hybrid tilapia infected with TiLV in Ayutthaya Province, Thailand, in 2021. To confirm the infection, the total RNA was extracted from the livers of the red hybrid tilapia using GENEzol^TM^ reagent (Geneaid Biotech, New Taipei City, Taiwan), and in line with a previous protocol [40], the TiLV RNA was detected using reverse transcription quantitative polymerase chain reaction (RT-qPCR) primers targeting TiLV segment 3. The extracted RNA subsequently served as the template for cDNA synthesis and the amplification of TiLV segment 4 (TiLV-S4) via PCR using specific primer pairs (forward primer 5′-GGATCCATATGGTGAGAACTACAAAGAC-3′ and reverse primer 5′-GTCGACTCGAGCTATCTTCCAACAGCCCC-3′). The primers were designed based on the sequence with GenBank accession number MK425013.1. The PCR product was cloned into the pET28a expression vector (Novagen, Tokyo, Japan). The pET28a–TiLV-S4 construct was subjected to DNA sequencing (Macrogen, Seoul, Republic of Korea). The recombinant TiLV segment 4 protein (rTiLV-S4) was expressed in the *Escherichia coli* strain BL21 (DE3) by induction with 1 mM isopropyl β-D-thiogalactopyranoside (Fermentas, Waltham, MA, USA) at 18 °C for 6 h while shaking at 225 rpm. The cell pellets were collected by centrifugation at 3000× *g* at 4 °C for 10 min and resuspended in a phosphate buffer (NaH_2_PO_4_ and Na_2_HPO_4_, pH 7.4) that contained 1 mM phenylmethylsulfonyl fluoride. The cell suspension was then sonicated using an XL2020 Sonicator Ultrasonic Processor XL (Misonix, Farmingdale, NY, USA). The crude protein containing the rTiLV-S4, as previously described [41], was collected from the supernatant after centrifugation at 12,000× *g* at 4 °C for 20 min. The concentration of the crude protein was determined using a bicinchoninic acid assay (Fermentas, Waltham, MA, USA).

### 2.2. Chicken Immunization and Egg Collection

To promote IgY production against TiLV, two hens were immunized with rTiLV-S4. The first antigen immunization contained 2 mg/mL of rTiLV-S4 in Freund’s complete adjuvant (Sigma-Aldrich, St. Louis, MO, USA) and was followed by the second and third boosters, which contained rTiLV mixed with Freund’s incomplete adjuvant (Sigma-Aldrich, St. Louis, MO, USA). The injections were administered at one-week intervals, with 1 mL of the mixture injected intramuscularly into three different sites in the pectoral musculature. Eggs were collected from the hens prior to immunization (T0) and one (T1) or two weeks (T2) after the last immunization and stored at 4 °C until use (Supplementary Figure S1). The procedures were approved by the Institutional Animal Care and Use Committee at Kasetsart University under protocol number ACKU65-VET-088. The principles of replacement, reduction, and refinement were followed to ensure the ethical and humane treatment of the chickens involved in this study.

### 2.3. Total IgY Extraction

The total IgY was extracted from the egg yolks using gradients of the polyethylene glycol (PEG) precipitation techniques described elsewhere [42,43,44]. The eggshell was carefully cracked, and the yolk was separated from the egg white. After removing the remaining egg white with filter paper, the yolk membrane was punctured with a pipette tip, and the yolk was transferred to a 50 mL tube. The yolk was mixed with phosphate buffered saline (PBS) at twice the volume of the yolk. Subsequently, 3.5% PEG 6000 (*w*/*v*) (Sigma-Aldrich, St. Louis, MO, USA) was added, and the mixture was vortexed for 10 min. This step separated the suspension into two fractions: one containing the yolk and fatty substances and the other a liquid phase with IgY and other proteins. The tubes were centrifuged at 13,000× *g* for 20 min at 4 °C. The supernatant was filtered through paper and transferred to a new 50 mL tube. Next, 8.5% PEG 6000 (*w*/*v*) was added to the tube, mixed by a brief vortexing, and centrifuged at 13,000× *g* for 20 min at 4 °C. The supernatant was discarded, and the pellet was dissolved in 10 mL PBS and 12% PEG 6000 (*w*/*v*). The IgY extract was dialyzed overnight in 0.1% NaCl, followed by an additional 3 h in PBS at 4 °C, before being stored at −20 °C for further analysis.

### 2.4. IgY Characterization by SDS-PAGE and Western Blot Analysis

We initially confirmed the presence of IgY in the egg yolks using sodium dodecyl sulfate-polyacrylamide gel electrophoresis (SDS-PAGE) with a Protean II electrophoresis system (Bio-Rad, Hercules, CA, USA) by following the discontinuous buffer system method [45]. The collected egg yolks were electrophoresed in a 15% resolving SDS-PAGE gel under reducing conditions at 120 V for 70 min, and the protein bands were visualized using a Coomassie blue staining solution (Bio-Rad, Hercules, CA, USA). The protein band sizes were determined using protein molecular marker standards (AccuProtein Chroma range 16–250 kDa, Enzmart Biotech, Bangkok, Thailand).

Western blot analysis was subsequently conducted to confirm the immunogenic property of anti-TiLV-S4 IgY against the purified virus. One microgram of TiLV, which had been purified using a sucrose gradient [46], was electrophoresed in 12% SDS-PAGE at 120 V for 80 min and transferred to a polyvinylidene fluoride membrane (Bio-Rad, Hercules, CA, USA) using a mini blot transfer system. The membrane was washed three times with 0.1% Tween-20 in PBS (PBS-T) and then incubated overnight at 4 °C with a blocking buffer comprising 3% bovine serum albumin (BSA) in PBS-T. Following three washes with PBS-T, the membrane was incubated with anti-TiLV-S4 IgY at a dilution of 1:100 for 1 h at room temperature. The membrane was washed again with PBS-T and incubated with a horseradish peroxidase-labelled goat anti-chicken IgY antibody (Abcam, Carlsbad, CA, USA) at a dilution of 1:2000 for 1 h at room temperature. After three final washes with PBS-T, the enhanced chemiluminescence substrate (Lumiflash^TM^, Visual Protein, Taipei, Taiwan) was applied and visualized using the ChemiDoc MP^TM^ Imaging System (Bio-Rad, Hercules, CA, USA).

### 2.5. In Vitro TiLV Neutralization Using Anti-TiLV-S4 IgY Antibodies

The anti-TiLV-S4 IgY antibody was serially diluted two-fold with Leibovitz’s L-15 medium (Sigma-Aldrich, St. Louis, MO, USA) to obtain dilutions of 1:2, 1:5, and 1:10. The prepared antibody was mixed with 10^3^ TCID_50_/mL of TiLV strain VET-KUTV08 at a ratio of 1:1. The mixture was incubated in an Eppendorf^®^ ThermoMixer C (Eppendorf, Hamburg, Germany) at 25 °C for 2 h with continuous shaking at 400 rpm. The positive control consisted of 10^3^ TCID_50_/mL TiLV mixed with an L-15 medium at a ratio of 1:1, and the negative control was the L-15 medium only. One hundred microliters of these mixtures and controls were added to a 96-well plate containing confluent E-11 cells, with four replicates per dilution. The cells were incubated at 25 °C for 1 h, then the mixtures were discarded, and a new L-15 medium supplemented with 2% FBS was added to each well. The cells were grown continuously for 7–12 days at 25 °C and monitored daily for the cytopathic effect (CPE) under an inverted microscope (CKX53, Olympus, Tokyo, Japan).

### 2.6. Virus Titration

To quantify the viral concentration following inhibition by the anti-TiLV IgY antibodies, the cells were initially lysed using a freeze–thaw process. The media from each replicate well of each dilution were pooled and transferred to 1.5 mL microcentrifuge tubes. The samples were centrifuged at 3000× *g* for 10 min at 4 °C. The supernatant was collected and serially diluted 10-fold to obtain dilutions ranging from 10^−1^ to 10^−8^. The diluted samples were added to 96-well plates that contained confluent E-11 cells with five replicate wells per sample. The plates were incubated at 25 °C for 1 h. Following incubation, the media in each well were replaced with an L-15 media supplemented with 2% FBS, and the plates were incubated at 25 °C for 7–12 days. The plates were observed daily for CPEs. The 50% tissue culture infectious dose (TCID_50_) was calculated using the Reed and Muench method [47].

### 2.7. Immunofluorescence Assay to Detect TiLV in the RHTiB Cell Line

An immunofluorescence assay (IFA) targeting TiLV was used to confirm the inhibition of viral entry into RHTiB cells (a cell line from the brain tissue of red hybrid tilapia) [48]. Briefly, the RHTiB cells were cultured in Leibovitz’s L-15 medium supplemented with 10% FBS, 100 U/mL penicillin, 0.1 mg/mL streptomycin, and 0.25 µg/mL amphotericin B at a pH of 7.4 at 25 °C without CO_2_. When the cells achieved 70% confluence, they were trypsinized, counted, and diluted to a concentration of 2 × 10^5^ cells/mL. Subsequently, 500 µL of the cell suspension was seeded onto cell culture slides (SPL Life Science, Pocheon-si, Gyeonggi-do, Republic of Korea) in an L-15 medium containing 10% FBS and incubated until 80–90% confluence was achieved. The cells were infected using different methods: 100 µL TiLV mixed with 100 µL L-15 medium (positive control), TiLV mixed with a 1:2 or 1:10 IgY solution, TiLV mixed with IgY at T0, control egg, and control L-15 medium (negative control). These mixtures were incubated at 25 °C with continuous shaking at 400 rpm for 2 h (Eppendorf^®^ ThermoMixer^®^ C, AG, Darmstadt, Germany). The cells were washed twice with an L-15 medium without FBS, incubated with the virus mixtures for 1 h, and subsequently replaced with an L-15 medium containing 2% FBS, followed by incubation at 25 °C for 24 h.

The cells were fixed with ice-cold 100% methanol for 10 min and washed twice with PBS. They were then treated with 0.3% Triton X-100 in PBS for 10 min and washed again with PBS. The membrane was blocked with 2% BSA in PBS for 30 min to prevent nonspecific binding, followed by overnight incubation at 4 °C with the primary antibody (IgG of TiLV) in a blocking solution at a 1:100 dilution. The cells were then incubated with the secondary antibody (goat anti-rabbit IgG H&L Alexa Fluor^TM^ 488; Abcam, Carlsbad, CA, USA) in PBS at a dilution of 1:500 for 1 h at room temperature and washed with PBS. The cell nuclei were stained with 4′,6-diamidino-2-phenylindole (DAPI; Sigma-Aldrich, St. Louis, MO, USA) at a 1:1000 dilution for 15 min, washed with PBS, mounted with ProLong^TM^ Gold Antifade reagent (Invitrogen, Thermo Fisher Scientific, Waltham, MA, USA) on glass slides, and then a cover glass was placed over them. All images were captured using a confocal microscope (Fluoview 3000, Olympus, Tokyo, Japan), which confirmed the specific binding of IgY to the TiLV-infected cells through the colocalization of the DAPI and Alexa Fluor signals. The fluorescence intensity values were quantified using cellSens Dimension software version 2.3 (Olympus, Tokyo, Japan). Five areas were randomly selected, and the fluorescence intensity of the green signal, which indicated positive TiLV-infected cells, was measured. The green signal intensity was analyzed as mean ± standard deviation (SD) and compared with the positive control, egg control, and TiLV mixed with the 1:2 or 1:10 IgY solutions.

### 2.8. Statistical Analysis

The viral titer and fluorescence intensity were presented as mean ± SD. The mean fluorescence intensity data between different IgY concentrations were assessed using one-way analysis of variance (ANOVA), and the TCID_50_ of the virus between different treatment and time points of infection was analyzed using two-way ANOVA. GraphPad Prism software version 8.0 was used in the analysis, and a *p*-value less than 0.05 was considered statistically significant.

## 3. Results

### 3.1. Immunization and Preparation of Chicken IgY

All the hens immunized with crude protein containing rTiLV-S4 via intramuscular injections remained healthy with no observed abnormalities or mortality throughout the study period. Furthermore, no signs of inflammation, adverse reactions, or pathological changes were detected at the injection sites. The IgY antibody purification process revealed protein bands at approximately 20 kDa and 60 kDa, which corresponded to light (LC) and heavy chains (HC) of immunoglobulin, respectively (Supplementary Figure S2). Additionally, an impurity band at approximately 35 kDa, identified as the C-terminal fragment of the vitellogenin II precursor, was also observed [49].

**Figure 1:**
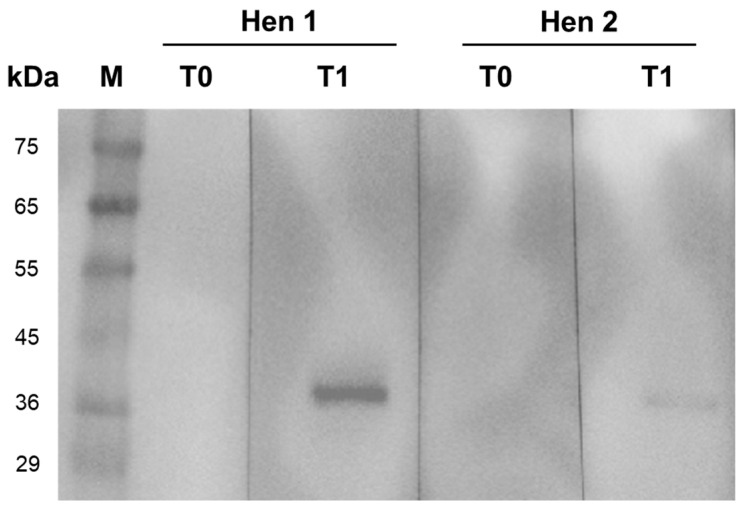
Western blot analysis showing specific binding of purified IgY from two individual chickens (Hen 1 and Hen 2) to the recombinant TiLV segment 4 protein (rTiLV-S4). No binding was observed at the pre-immunization stage (T0). However, distinct bands corresponding to rTiLV-S4 at 38 kDa were detected one-week post-immunization (T1) in the samples from both hens. Lane M represents the protein marker.

The specific binding of the purified IgY antibodies against rTiLV-S4 extracted from the eggs of two hens was evaluated using Western blot analysis at T0 and T1 (Figure 1). The analysis confirmed the presence of specific binding in the post-immunization samples, which was indicated by a distinct 38 kDa band corresponding to TiLV. Notably, the binding signal was stronger in the samples from the first hen (Hen 1) compared to the second hen (Hen 2). Importantly, no nonspecific binding was observed in any of the samples, which suggested the specificity of the purified IgY antibodies.

### 3.2. Neutralization of IgY Against TiLV

**Figure 2:**
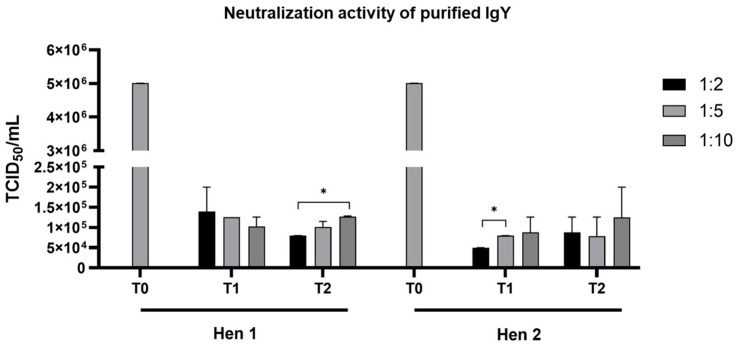
Neutralization activity of purified IgY against rTiLV-S4, assessed by the 50% tissue culture infective dose (TCID_50_)/mL. The E-11 cells treated with purified IgY from the eggs of two hens (Hen 1 and Hen 2) collected after one week (T1) and two weeks (T2) post-immunization and infected with TiLV showed significantly lower infectivity compared to the control group (pre-immunization). Bars represent the mean ± standard deviation (SD) of the measured neutralization activity. * *p* < 0.05.

The neutralization activity of the purified IgY dilutions obtained from the eggs of the two hens at T1 and T2 post-immunization was evaluated at various dilutions (1:2, 1:5, and 1:10) against TiLV infectivity in E-11 cells (Figure 2). All the E-11 cells pretreated with anti-TiLV-S4 IgY, irrespective of the dilution, exhibited significantly reduced TiLV infection levels compared to the positive control, which had an infectivity of 5.01 × 10^6^ TCID_50_/mL. Of note, no statistical differences in TiLV infectivity were apparent between the cells neutralized with IgY from the T1 and T2 groups of either hen. For the IgY purified from Hen 1 at T2, the 1:2 dilution resulted in a significantly lower infectivity level of 7.94 × 10^4^ TCID_50_/mL compared to the 1:10 dilution, which yielded 1.26 × 10^5^ TCID_50_/mL. Similarly, for the IgY purified from Hen 2 at T1, the 1:2 dilution achieved a TiLV infectivity level of 5.01 × 10^4^ TCID_50_/mL, which was significantly lower than the 7.94 × 10^4^ TCID_50_/mL observed with the 1:5 dilution.

**Figure 3:**
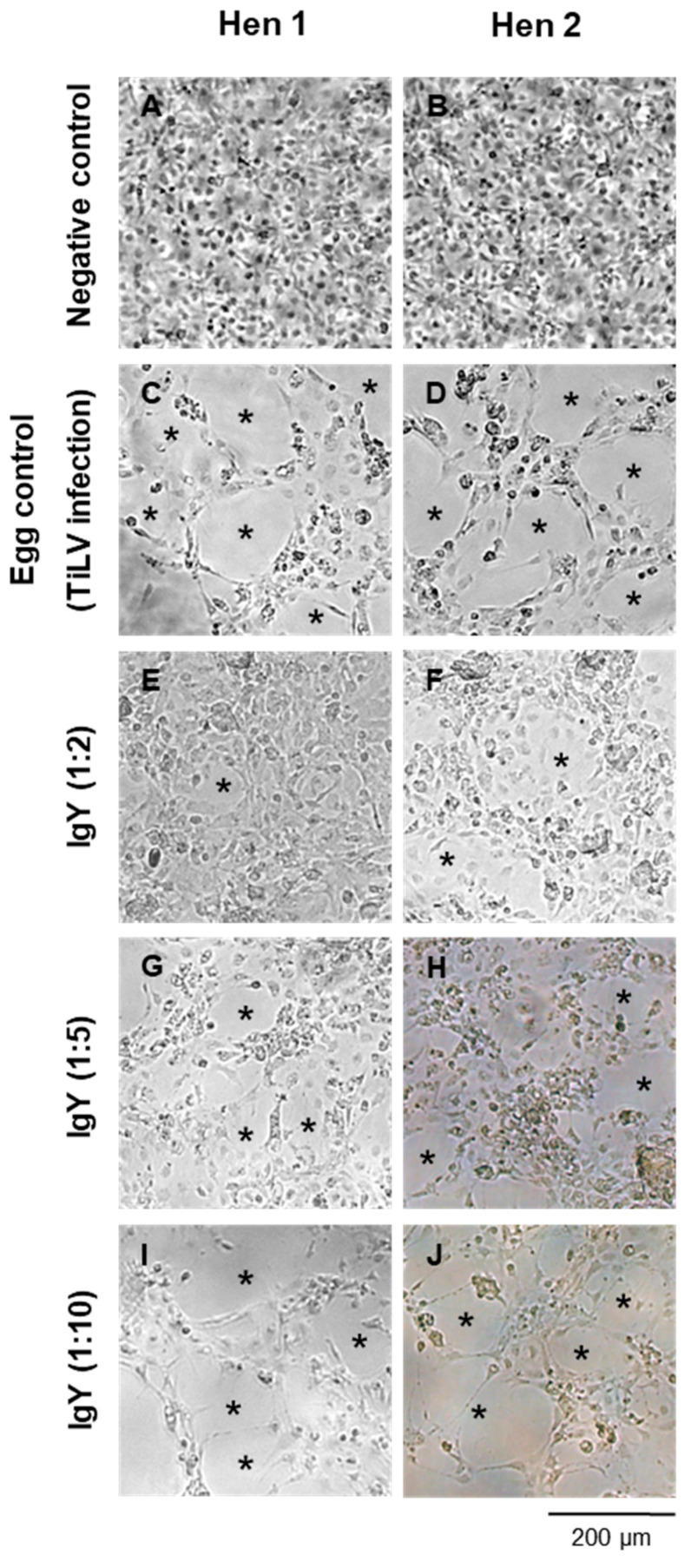
Evaluation of the neutralization activity of purified IgY from Hen 1 and Hen 2 against TiLV infectivity in E-11 cells at 7 days postinfection. The cellular morphologies are shown. (**A**,**B**) Negative control cells; (**C**,**D**) TiLV-infected cells treated with pre-immunized IgY (egg control); (**E**,**F**) infected cells treated with purified IgY at a 1:2 dilution; (**G**,**H**) 1:5 dilution; (**I**,**J**) 1:10 dilution. Asterisks (*) indicate the presence of cytopathic effects (CPE). Scale bar: 200 µm (10× magnification).

The neutralization activity of the purified IgY against TiLV was further assessed based on the presence of the CPE in the E-11 cells (Figure 3). In comparison to the uninfected E-11 cells, which exhibited no signs of the CPE (Figure 3A,B), the cells inoculated with TiLV as a positive control displayed a noticeable CPE at 7 days postinfection (dpi) (Figure 3C,D). When TiLV was incubated with IgY at different dilutions, a dose-dependent neutralization effect was observed. Specifically, the lowest level of CPE was detected in the group treated with IgY diluted at a ratio of 1:2, which demonstrated the highest neutralization efficiency (Figure 3E,F). In contrast, the cells treated with IgY diluted at a ratio of 1:5 showed an increased level of the CPE (Figure 3G,H), while those treated with IgY diluted at a ratio of 1:10 had CPE levels comparable to those of the positive control (Figure 3I,J). Based on these findings, the IgY purified from Hen 2 at T1 with a 1:2 dilution demonstrated the lowest TiLV infectivity in the E-11 cells and was selected for further study.

### 3.3. Immunofluorescence Assay

**Figure 4:**
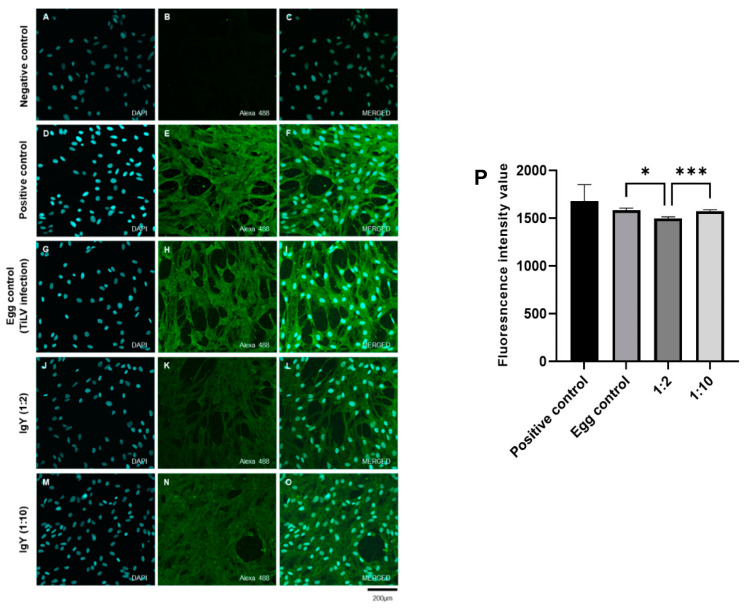
Neutralization activity of purified IgY in reducing TiLV antigen levels in RHTiB cells assessed using an immunofluorescence assay (IFA). (**A**–**C**) Negative control cells demonstrate only blue 4′,6-diamidino-2-phenylindole (DAPI) staining. (**D**–**F**) TiLV-infected cells (positive control) show colocalization of the TiLV antigen (green fluorescence) within the cytoplasms of the RHTiB cells. (**G**–**I**) Egg control group, namely RHTiB cells treated with purified IgY extracted from egg yolk during the pre-immunization period. (**J**–**L**) RHTiB cells treated with purified IgY at a 1:2 dilution and infected with TiLV. (**M**–**O**) RHTiB cells treated with purified IgY at a 1:10 dilution and infected with TiLV. (**P**) Graph showing the mean fluorescence intensity (MFI) values as mean ± SD for the positive control, egg control, and IgY-treated groups (1:2 and 1:10 dilutions). Statistically significant differences are indicated as * *p* < 0.05 and *** *p* < 0.001.

The neutralizing activities of the purified IgY in inhibiting TiLV entry into the RHTiB cells were further evaluated using an immunofluorescence assay (Figure 4). In comparison to the uninfected control cells, which showed no green fluorescence signals (Figure 4A–C), the TiLV-infected cells without IgY treatment (positive control) showed strong green fluorescence within the RHTiB cells at 1 dpi (Figure 4D–F). The infected cells incubated with the IgY samples from the pre-immunization period (egg control) exhibited similar levels of the green fluorescence signal (Figure 4G–I). Interestingly, treatment with the purified IgY remarkably reduced the presence of TiLV in the RHTiB cells. The cells treated with IgY at a dilution of 1:2 demonstrated a substantial reduction in green fluorescence intensity compared to the positive control (Figure 4J–L). However, although the cells treated with IgY at a 1:10 dilution showed higher fluorescence intensity relative to the 1:2 dilution, the intensity remained considerably lower than that of the positive control (Figure 4M–O).

When comparing the mean fluorescence intensity (MFI) values among the positive control, egg control, and IgY-treated groups (dilutions of 1:2 and 1:10), the positive control showed the highest MFI (1681 ± 171.5), followed by the egg control (1586 ± 19.78). The group treated with IgY at a 1:2 dilution showed an MFI of 1497 ± 17.18, while the group treated with IgY at a 1:10 dilution had an MFI of 1576 ± 13.59. Further statistical analysis revealed that the cells treated with IgY at a 1:2 dilution had significantly lower MFI values compared to the egg control and cells treated with IgY at 1:10 dilution groups (* *p* < 0.05 and *** *p* < 0.001, respectively) (Figure 4P).

## 4. Discussion

Infection with TiLV causes serious disease in tilapia and poses a major challenge to global tilapia aquaculture. Various strategies are being explored to prevent the spread of infection and reduce the impacts of TiLV disease. Current approaches to TiLV mitigation include vaccine development [41], the application of probiotics [50], and the use of feed additives [51]. However, these strategies either require prolonged development and regulatory approval or may not provide immediate protection during outbreaks. Passive immunization using IgY offers a complementary approach characterized by noninvasive production, high specificity, cost effectiveness, and minimal ethical concerns. These attributes position IgY as a valuable tool for enhancing biosecurity and sustainability in aquaculture [28]. In this study, we successfully generated TiLV-specific IgY antibodies by immunizing laying hens with the rTiLV-S4 protein expressed in *E. coli* [41]. The immunization protocol involved the intramuscular administration of rTiLV-S4 formulated with Freund’s complete adjuvant for the initial dose, followed by booster doses with Freund’s incomplete adjuvant [44]. A significant increase in IgY levels was detected in the eggs of the hens immunized with the antigen, thereby confirming that this immunization strategy effectively stimulated robust IgY production, which is consistent with the findings of previous studies [52]. The specificity of the purified IgY was confirmed by Western blot analysis, which demonstrated strong binding to the TiLV-S4 without nonspecific interactions. Upon antigen exposure, the immune systems of the hens produced specific antibodies, which were then transferred to the egg yolks [29]. To purify these IgY antibodies, the egg yolks were subjected to PEG precipitation, a widely used and cost-effective method for large-scale antibody extraction [53]. This process effectively removed lipids and enriched the IgY concentration, which ensured antibody stability and solubility [54]. Optimized PEG precipitation techniques have been shown to improve IgY purity and yield, which supports the feasibility of IgY for broader aquaculture applications [55]. In our study, both HCs and LCs were detected, and a specific band demonstrating the interaction between the antibody and rTiLV-S4 was confirmed. After PEG precipitation, the neutralizing activity of the purified IgY against TiLV was proved in a continuous cell line. Interestingly, neutralization assays using different concentrations of IgY revealed the dose-dependent inhibition of TiLV, with the highest antiviral efficacy at a 1:2 dilution. These findings are consistent with previous studies demonstrating the dose-dependent effects of IgY-mediated inhibition on viral and bacterial pathogens in fish [21,39]. In particular, our study showed that IgY antibodies effectively reduced the viral load and mitigated CPE, which highlights their potential as a therapeutic approach against this emerging virus. Although variations in infectivity were detected when tissue was treated with IgY from different hens, its ability to reduce the CPE in TiLV-infected E-11 cells remained consistent, which highlights the antiviral potential of IgY. It should be considered that the variation in neutralization efficiency among the IgY samples may have been influenced by individual differences in immune responses to the same antigenic stimulation. Factors such as genetic background, age, breed, and egg-laying capacity have been reported to influence antibody production in laying hens [23,29]. These findings emphasize the need for further optimization of immunization protocols to ensure the consistency and potency of IgY antibodies against TiLV.

The neutralization activity of IgY was further confirmed using an immunofluorescence assay, which demonstrated a significant reduction in TiLV antigens in RHTiB cells, with the highest inhibition observed at a 1:2 dilution of purified IgY. Mechanistically, IgY may inhibit viral replication by blocking viral entry into host cells through specific binding to viral proteins, thereby preventing their attachment to the host cell receptors [38,56]. This evidence supports the potential of IgY as an effective antiviral strategy for controlling TiLV infection. Despite these promising findings, however, challenges remain in optimizing IgY deployment for field applications. Effective implementation in aquaculture settings requires key factors, such as optimal fish size, dosage, and delivery methods, to be addressed [23]. Such factors must be thoroughly researched and validated before IgY can be adopted for use in fish farms. For example, while the administration methods of intraperitoneal and intramuscular injections ensure systemic immunity, they are labor-intensive and impractical for large-scale aquaculture operations [57]. As a noninvasive alternative, oral administration may be suitable for targeting gastrointestinal and systemic pathogens; however, its efficacy may be limited by enzymatic degradation in the digestive tract [58]. Recent advancements in encapsulation technologies, such as chitosan–alginate microcapsules, have improved IgY stability and bioavailability, which suggests the future feasibility of oral delivery for large-scale aquaculture applications [59]. Therefore, encapsulating IgY in coated feed pellets or specialized formulations to enhance its stability and protect against enzymatic degradation presents a promising approach for effective oral delivery in fish farming. Nevertheless, further research is needed to establish standardized protocols for TiLV-specific IgY production and delivery to ensure consistency, efficacy, and practical implementation in the management of TiLV infection using IgY.

## 5. Conclusions

Passive immunization with IgY presents a sustainable and ethical approach to disease control in aquaculture. In our study, we demonstrated the potential application of IgY antibodies to mitigate TiLV infections in tilapia. The rTiLV-S4-specific IgY antibodies derived from laying hens had a dose-dependent neutralization effect and significantly reduced viral infectivity and the CPE in E-11 cells. Immunofluorescence assays further confirmed the inhibitory properties of IgY and revealed its ability to prevent TiLV infection in RHTiB cells. Despite these advantages, challenges remain in optimizing IgY deployment, particularly in determining the most effective administration routes and ensuring long-term stability. Further studies should focus on developing scalable application strategies to maximize the impact of IgY immunization on disease prevention in commercial fish farming.

## Data Availability

The data presented in this study are available on request from the corresponding author. The data are not publicly available due to the anonymity granted to all participating parties.

## Supplementary Materials

The following supporting information can be downloaded at https://www.mdpi.com/article/10.3390/v17030448/s1, Figure S1: The schematic illustrates the immunization protocol and egg collection schedule for the production of chicken egg polyclonal IgY against tilapia lake virus (TiLV); Figure S2: Gel electrophoresis of the IgY samples purified using polyethylene glycol (PEG) precipitation at different stages.
