# Supplementary material for: Development of IgY-Based Passive Immunization Against Tilapia Lake Virus: Development and In Vitro Neutralization Assays
Source: Viruses. 2025 Mar 20;17(3):448. doi: 10.3390/v17030448 (PMC11946193; doi:10.3390/v17030448)
Supplement: Supplementary file 1 [file viruses-17-00448-s001.zip › viruses-3532519-supplementary.pdf]

Supplementary Materials:

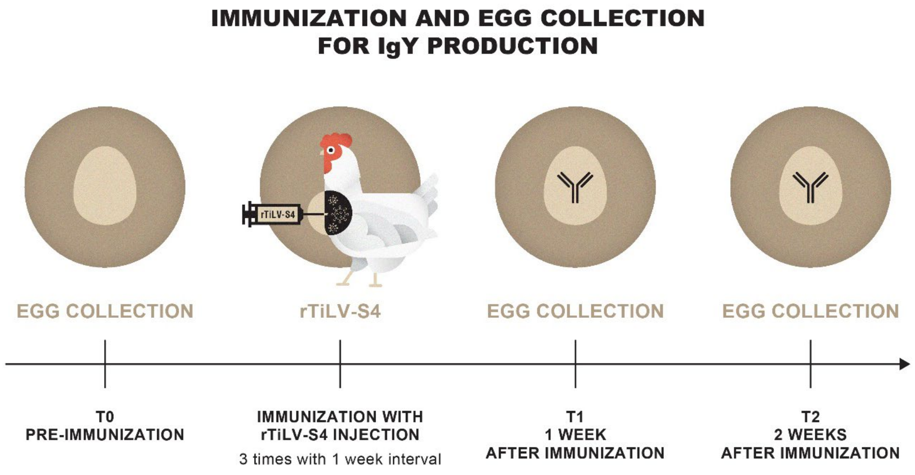

Figure S1: The schematic illustrates the immunization protocol and egg collection schedule for the production of chicken egg polyclonal IgY against tilapia lake virus (TiLV).

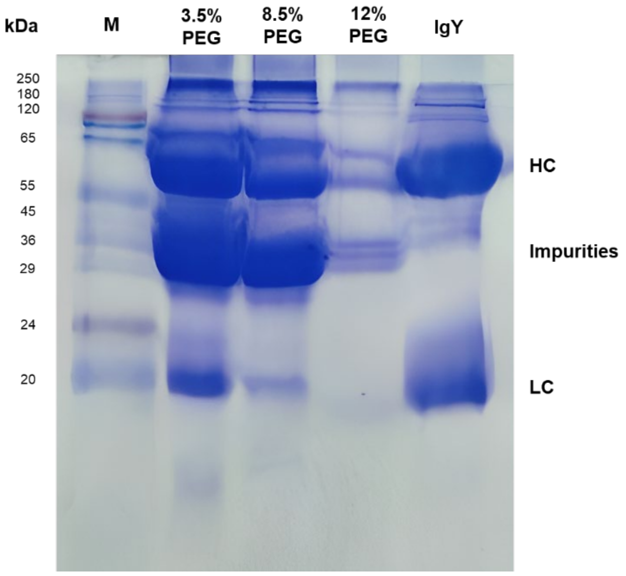

Figure S2: Gel electrophoresis of the IgY samples purified using polyethylene glycol (PEG) precipitation at different stages. Lane 1: Protein molecular weight marker (M); Lane 2: Purified IgY following 3.5% PEG precipitation; Lane 3: Purified IgY following 8.5% PEG precipitation; Lane 4: Purified IgY following 12% PEG precipitation; Lane 5: Purified IgY following dialysis. The protein bands observed at approximately 60 kDa and 20 kDa correspond to heavy (HC) and light chains (LC) of IgY, respectively. A protein band at approximately 35 kDa indicates impurities identified as the C-terminal fragment of the vitellogenin II precursor.
